# Access to quality care after injury in Northern Malawi: results of a household survey

**DOI:** 10.1186/s12913-023-10521-8

**Published:** 2024-01-24

**Authors:** John Whitaker, Abena S. Amoah, Albert Dube, Rory Rickard, Andrew J. M. Leather, Justine Davies

**Affiliations:** 1 Institute of Applied Health Research, University of Birmingham, Birmingham, UK; 2 King’s Centre for Global Health and Health Partnerships, School of Life Course and Population Sciences, Faculty of Life Sciences and Medicine, King’s College London, London, UK; 3 Academic Department of Military Surgery and Trauma, Royal Centre for Defence Medicine, Birmingham, UK; 4 Malawi Epidemiology and Intervention Research Unit (Formerly Karonga Prevention Study), Chilumba, Malawi; 5 Department of Population Health, Faculty of Epidemiology and Population Health, London School of Hygiene & Tropical Medicine, Keppel Street, London, UK; 6 Department of Parasitology, Leiden University Center for Infectious Diseases, Leiden University Medical Center, Leiden, the Netherlands; 7 Medical Research Council/Wits University Rural Public Health and Health Transitions Research Unit, Faculty of Health Sciences, School of Public Health, University of the Witwatersrand, Johannesburg, South Africa; 8 Department of Global Surgery, Stellenbosch University, Stellenbosch, South Africa

**Keywords:** Wounds and injuries, Health services research, Health care surveys, Health care quality, access, and evaluation, Malawi

## Abstract

**Background:**

Most injury care research in low-income contexts such as Malawi is facility centric. Community-derived data is needed to better understand actual injury incidence, health system utilisation and barriers to seeking care following injury.

**Methods:**

We administered a household survey to 2200 households in Karonga, Malawi. The primary outcome was injury incidence, with non-fatal injuries classified as major or minor (> 30 or 1–29 disability days respectively). Those seeking medical treatment were asked about time delays to seeking, reaching and receiving care at a facility, where they sought care, and whether they attended a second facility. We performed analysis for associations between injury severity and whether the patient sought care, stayed overnight in a facility, attended a second facility, or received care within 1 or 2 h. The reason for those not seeking care was asked.

**Results:**

Most households (82.7%) completed the survey, with 29.2% reporting an injury. Overall, 611 non-fatal and four fatal injuries were reported from 531 households: an incidence of 6900 per 100,000. Major injuries accounted for 26.6%. Three quarters, 76.1% (465/611), sought medical attention. Almost all, 96.3% (448/465), seeking care attended a primary facility first. Only 29.7% (138/465), attended a second place of care. Only 32.0% (142/444), received care within one hour. A further 19.1% (85/444) received care within 2 h. Major injury was associated with being more likely to have; sought care (94.4% vs 69.8% *p* < 0.001), stayed overnight at a facility (22.9% vs 15.4% *P* = 0.047), attended a second place of care (50.3% vs 19.9%, *P* < 0.001). For those not seeking care the most important reason was the injury not being serious enough for 52.1% (74/142), followed by transport difficulties 13.4% (19/142) and financial costs 5.6% (8/142).

**Conclusion:**

Injuries in Northern Malawi are substantial. Community-derived details are necessary to fully understand injury burden and barriers to seeking and reaching care.

**Supplementary Information:**

The online version contains supplementary material available at 10.1186/s12913-023-10521-8.

## Key messages

• *What is already known on this topic* – Empirical data on injury burden from low-income country settings is lacking, particularly from community derived sources.

• *What this study adds* – We conducted a household survey in Northern Malawi to understand incidence, health system utilisation, time delays and barriers to receiving care following injury. We found the burden of injuries was substantial with a community preference to attend primary facilities directly following injury, many not receiving care within 2 hours of injury. Almost a quarter of those injured did not seek care at all revealing important health system barriers inhibiting timely access to care.

• *How this study might affect research, practice, or policy* – Addressing the identified barriers to seeking timely care and streamlining care to the most appropriate facilities have important policy implications for injury care system strengthening in this and similar settings.

**Supplementary Information:**

The online version contains supplementary material available at 10.1186/s12913-023-10521-8.

## Background

The consequences of injury are broad. The World Health Organisation (WHO) has conceptualised a population’s injury burden pattern as a pyramid [1]. Fatal injuries are the tip, followed by injures requiring hospitalisation, emergency facility visits, and those outside of, or not reaching, the formal health system. Fatal injury accounts for 8% of global deaths [2] with most (90%) occurring in Low and Middle Income Countries (LMICs) [3]. The burden of non-fatal injuries is similarly important and substantial. One billion people sustain an injury sufficiently severe to require formal healthcare each year [4]. Trauma is responsible for 9.8% of all Disability Adjusted Life Years (DALY) globally and 22.0% of DALYs for young adult males [5]. This disease burden is also not uniformly distributed with DALY rates for children ninefold higher in sub-Saharan Africa compared to high-income countries [4]. Injuries also potentially place an enormous burden upon health systems. Up to 38% of surgically treatable disease burden in LMICs is injury-related [6, 7]. Nearly half of injury-related mortality occurs in individuals aged 15 to 44 during their most economically productive years [8, 9].

However, much of this injury data is from modelling, and empirical data is lacking. The WHO found that for reported injury-related DALYs in LMICs, 52% (74/144) of LMICs lacked complete, country-specific data for these calculations and used modelled estimates [8]. The development of trauma registries for prospective collection of trauma patient data supports surveillance and care improvements in HIC settings [10]. Whilst there are examples of successfully established registries in LMICs [11, 12], resource constraints have limited their widespread adoption [13]. Trauma registries are also limited to only recording patients with injuries treated in a facility and will not include those who choose not to seek care or do not survive to reach care. The lack of routine injury surveillance data in LMICs has led to calls for the adoption of non-traditional data sources to help fill this knowledge gap, such as demographic health surveillance and household surveys [14].

Within Malawi, injuries are estimated to account for 19% of non-communicable disease and injury disability-adjusted life years and 82% of injury burden affects those under 40 [15]. One third of road traffic collisions cause a fatality, with an average age of 32, with death more likely following collisions in rural areas, where the majority of Malawian’s live [16, 17]. However, obtaining reliable data on injuries is a challenge. The quality of routinely collected medical records in Malawi is insufficient to serve this purpose [16, 18]. Trauma registries exist in only a few facilities within Malawi, in Lilongwe and Blantyre, and not within the Northern region [12, 19]. Additionally, most studies on injury care within Malawi have focused on facility-based care. There is limited evidence available about burden of injuries and population barriers to seeking care following injury, in keeping with the paucity of global literature on this subject [20].

Household surveys can provide valuable information on nonfatal injury epidemiology and healthcare utilisation. Household surveys represent a valuable method to obtain generalisable population based data particularly where routine registration of injury data is absent [21]. Community-based studies include injuries that never reach facilities [22–26], and can complement facility based registry data that tends to dominate trauma epidemiology studies. We conducted a community-based household survey to investigate non-fatal injury burden and healthcare utilisation in Northern Malawi to better understand this neglected community-based phase of the post injury health system response.

Our objectives were:

1. To understand the incidence of injuries suffered by residents of the HDSS in the past 12 months
2. To explore health system utilisation including facility attendance and type following non-fatal injury
3. To establish time delays following non-fatal injury to receiving medical treatment in a facility.
4. To establish barriers to seeking care following non-fatal injury.

## Methods

We conducted a cross-sectional household survey in the Health and Demographic Surveillance System (HDSS) in Karonga, Northern Malawi between October 2019 and February 2020.

### Survey development

The survey was adapted from the WHO Guidelines for conducting community surveys on injuries and violence [21]. Additional questions were included to capture health-seeking behaviour, experience of, and reasons for, delays to seeking, reaching and receiving healthcare, and perceived healthcare quality. The survey was translated into the vernacular language of Chitumbuka by trained native speakers. Fieldworkers were trained and the survey was piloted within the local community on a sample of households including individuals with recent injury.

### Study setting

This study was conducted in the health system serving the HDSS population, Karonga District, representative of populations in Northern Malawi and similar contexts [27]. The HDSS population exceeds 40,000 and have participated in surveys for over twenty years [27]. The population is mostly rural, with 15% living in semi-urban settlements. The rural lakeshore district of Karonga is a subsistence economy reliant on farming and fishing [27]. A major paved road runs through the district but secondary roads are mostly unpaved [27].

Several primary health facilities serve the Karonga HDSS population including those run by the government, a military facility accessible by civilians, private facilities, and Christian Health Association of Malawi (CHAM) facilities. Two main secondary care facilities serve the population including a government facility approximately 70 km to the North and a CHAM facility approximately 40 km to the South over difficult terrain. Tertiary care is provided in the regional capital Mzuzu, approximately 150 km to the South at a government central hospital. Secondary and tertiary facilities provide resuscitative surgery for severe injuries.

### Sample size

We based our sample size calculation on surveying at least 100 individuals who had accessed care to achieve our study objectives related to health system access [28–31]. We assumed a non-fatal injury annual incidence of approximately 5% [28–30]. A sample of 10,000 persons was required to detect this proportion with a confidence interval id 0.046–0.055. Households within our study HDSS population are known to contain five individuals on average meaning 2,000 household surveys were required to cover the injury experience of 10,000 individuals. To adjust for non-response, experienced HDSS staff estimated 10% of local households might refuse or be unavailable to take part in the survey. Therefore, we estimated we required to sample 2,200 households to achieve our study objectives.

### Sampling

We used computer random number generation within Microsoft Excel for simple randomisation of all households within the HDSS to identify the sample for survey.

### Identification of participants

Any household member present aged over 18 was eligible to act as a proxy informant to complete the survey on behalf of the household. If no such household member was present or identified nearby following two attempts to visit the household was marked as missing. If more than one willing adult over 18 was present during the visit, the one most knowledgeable about recent injuries within the household was selected.

### Data collection

Data were collected by experienced and trained data collectors who administered the survey to participants in the local language. Field workers, some of whom were health care workers, were MEIRU staff members with extensive prior experience administering surveys in the community. Specific training on administering this survey, along with piloting amongst community members known to have had a recent injury, took place over a 1 week period. Data was collected into REDCap [32] using the mobile application on password-protected tablets. Data was uploaded to a central secure server daily. Respondents were asked if anyone in their household had suffered a fatal or non-fatal injury in the preceding 12 months, and if so, how many people had suffered an injury. Injury was defined as physical damage following any external force. A non-fatal injury minimum severity was defined as preventing the injured person from performing their usual activities for at least one day or seeking healthcare. If an individual had suffered more than one injury in the preceding 12 months, only the most severe injury, defined as that with the largest number of days unable to perform usual activities, was recorded.

For households with at least one non-fatal injury reported, the following details were collected for each injured person: the sex and age of the injured person in years; how long in total they were not able to perform normal activities in days; and whether the injured person sought medical attention or treatment outside of the household. If the answer to seeking medical attention or treatment outside the household was yes, questions about health system utilisation were asked. If the answer was no, questions about reasons for not seeking care were asked.

Questions asked of respondents who indicated the injured person had sought medical treatment, were: where care was first sought (named facility or other specified with free text) and whether a second facility was attended, and if yes where (named facility or other specified with free text). Time delays for care seeking were asked as follows: how long after the injury was the decision taken to seek care; how long it took to reach the first care facility after deciding to seek care; and how long it took to receive treatment once at the first facility (exclusive categories of < 1 h, 1–2 h, 2–4 h, 4–6 h, 6–12 h, 12–24 h, > 24 h). The delay in receiving treatment was the time between arriving at a facility and actually receiving something considered treatment from a healthcare worker, such as medications, IV fluids or wound care. Respondents were further asked how satisfied the treated person was that the care received was good quality (exclusive categories of very satisfied, satisfied, neither satisfied nor dissatisfied, dissatisfied, or very dissatisfied) and whether the injured person stayed overnight in a health facility and if yes for how many days.

Respondents who indicated the injured person had not sought medical treatment were asked the reasons for this decision (able to select multiple categories from “The injury was not serious enough to need medical care”, “The person or family had other priorities or responsibilities”, “It was too difficult to get transport to health facility”, “The family member responsible for decisions about seeking care did not want the injured person to seek care”, “The financial cost of seeking care was too much”, “The health facility was too far away”, “The injured person prefers to see traditional healers for health problems”, “The health facility would not provide effective treatment for this problem”, “The injured person did not believe that is was right to seek care following an injury”, “The health facility would not treat the injured person with respect”, “The injured person did not know healthcare was available”, “People fear the consequences of helping an injured person (e.g. being accused of causing the injury)”, “The health facility would not communicate well with the injured person and family” and other specified with free text). From the same reasons, the most important was also identified by participants.

### Outcome variables

The primary outcome for this analysis is the incidence of at least one injury in the previous 12 months, stratified by major and minor for non-fatal injuries. As is common in household studies on nonfatal injuries [33], injuries were categorised as major or minor based on the number of days of disability. Injuries with an associated disability of between 1 and 29 days are minor, those with 30 days or more are major. The number of occupants for households surveyed (incidence denominator) was taken from available HDSS surveillance data. Secondary outcomes are patterns of facility preference and use, time delays to seeking, reaching and receiving care, perceived care quality and barriers preventing seeking care.

Facilities named were categorised as “government primary”, “government secondary”, “government tertiary”, “faith-based primary”, “faith-based secondary”, “military primary”, “private primary” and “unknown”. Facilities were further categorised into facility type as primary or referral (including secondary and tertiary care). Time delays to care were classified into receiving care in less than the “Golden Hour” (seeking, reaching and receive care all took < 1 h), less than the Lancet Commission on Global Surgery (LCoGS) 2-h target but more than 1 h (two of seeking, reaching or receiving care took < 1 h and the other took 1–2 h), and longer than 2 h (any other combination of delay times). Both the “Golden” 1 h target and LCoGS 2 h targets for time to key patient interventions following injury are recognised internationally as a benchmark for high quality health systems to aim for [34–37]. Free text responses for other specified answers were reviewed. Where suitable these were assigned to an existing category or a new category was assigned.

### Analysis

Counts, percentages, median and interquartile ranges, describe the results. For those sustaining an injury, using SPSS, we performed univariable analysis using chi-squared (or fisher’s exact if cell counts < 5) to test for associations between sustaining a major injury rather than minor injury and whether the patient, sought care or not, stayed overnight in a facility or not, attended a second place of care or not, first facility type visited and whether they received care within 1 or 2 h from injury or not. Missing or non response is reported.

### Ethical considerations

All study details were explained to all study participants. They were provided with a participant information leaflet and consent form which they read or was read out to them in Chitumbuka by a native speaking research assistant. Any questions about the study conduct were answered, and participants signed or provided a thumbprint on an informed consent form, confirming their agreement to participate in the study. This was witnessed. Therefore, informed consent was obtained from all subjects. The study was approved by the Malawi National Health Sciences Research Committee (ref 19/07/2368) and the UK MOD Research and Ethics Committee (ref 961/MODEC/19). All methods were carried out in accordance with relevant guidelines and regulations.

### Patient and public involvement

A community sensitization meeting took place. Traditional heads were invited to attend a meeting where all aspects of the community study was explained, and questions answered. This is routine practice on the introduction of new studies within the MEIRU Karonga HDSS [27].

## Results

**Fig. 1:**
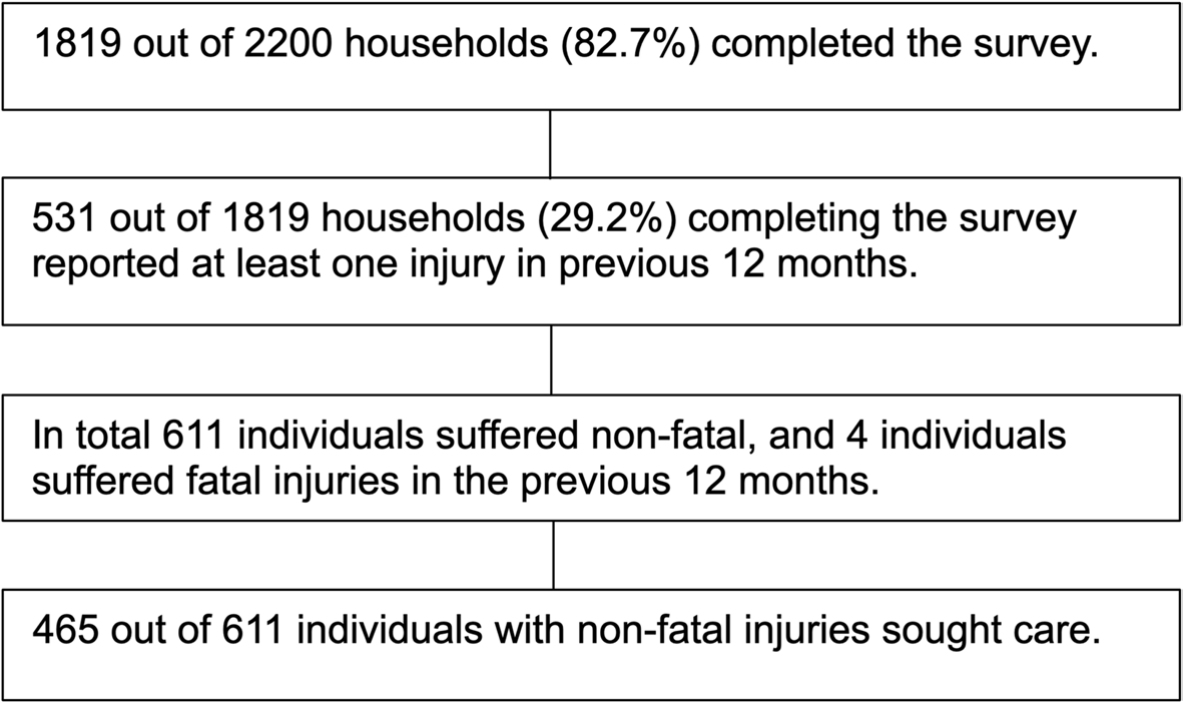
Flow diagram of household participant responses for injury incidence and healthcare seeking

**Fig. 2:**
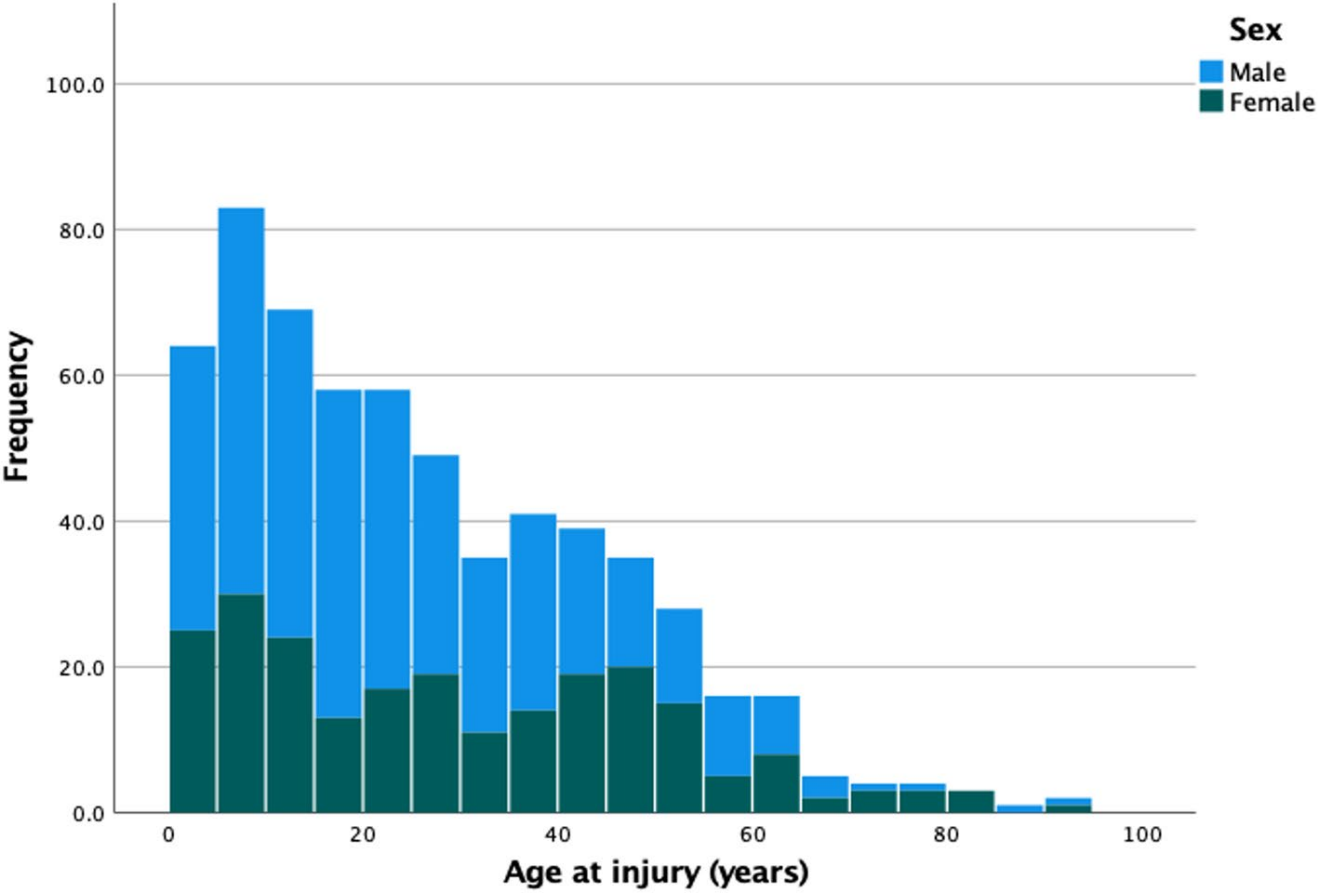
Household survey histogram of injured patients according to age at injury and sex. Bars represent 5-year intervals, male sex in blue and female sex in green

Out of 2200 households visited, 82.7% (1819/2200) completed the survey (Fig. 1). Over a quarter of households, 29.2% (531/1819), reported at least one member sustaining at least one injury in the previous 12 months. These 531 households reported 611 non-fatal injuries and four fatal injuries in the preceding 12 months. This represented an incidence of 6900 per 100,000 (615/8866). Most non-fatal injuries, 73.4% (447/609), were minor (< 30 days disability) whilst 26.6% (162/609) were major (30 days disability or more). Disability days was missing for 2 individuals. Males represented 61.9% (378/611) of those injured, and the median age at time of injury was 22 years (IQR 10–40) (Fig. 2).

**Fig. 3:**
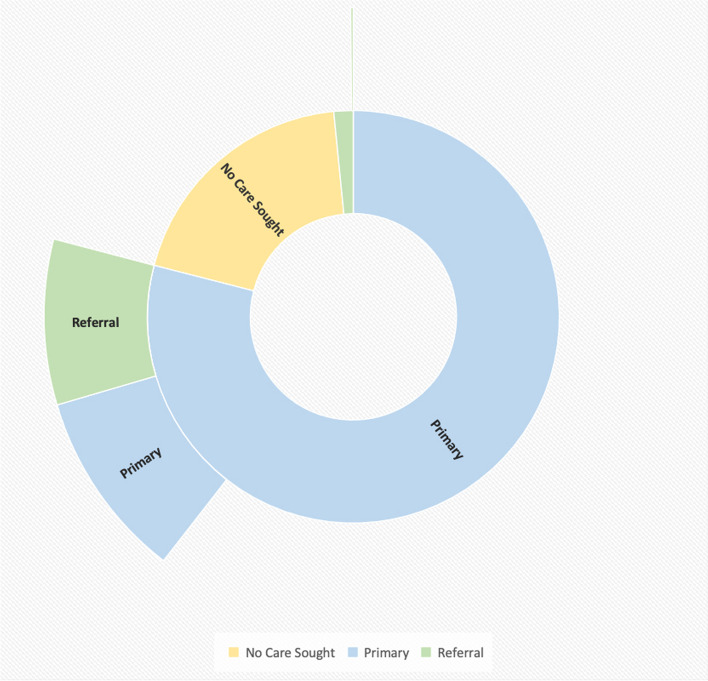
Household survey sunburst chart showing summary (primary blue, referral green, no care sought yellow) of first facility type (innermost ring) and second facility type (outer ring) attended (where applicable)

Three-quarters of those injured, 76.1% (465/611), reported seeking medical attention after the injury. Almost all, 96.3% (448/465), attended a primary facility first. This was most commonly a primary government facility (67.7%, 315/465) followed by private primary facilities (13.1%, 61/465) (Table 1). Almost a third of those seeking care, 29.7% (138/465), reported attending a second place of care following the injury. For 43.9% (61/138), this was the district secondary care referral hospital. The pattern of facility attendance by facility type is shown in Fig. 3.

**Table 1:** Household survey demographics, health facility care seeking and satisfaction according to injury severity

	All	Minor severity	Major severity
Age in years (median)	22.0	21.0	32.5
	All % (n/610)	Minor severity %(n/446)	Major severity %(n/162)
Male sex	62.0 (378)	61.2 (273)	64.0 (103)
	All % (n/611)	Minor severity %(n/446)	Major severity %(n/162)
Sought care after injury	76.1 (465)	70.0 (312)	94.4 (153)
Where did the injured person first seek medical treatment for his/her injury? By facility type^a^	All % (n/465)	Minor severity %(n/311)	Major severity %(n/153)
Primary any	96.3 (448)	95.2 (296)	98.7 (151)
Secondary any	1.9 (9)	2.3 (7)	1.3 (2)
Tertiary	0.2 (1)	0.3 (1)	0 (0)
Other	1.5 (7)	2.3 (7)	0 (0)
Where did the injured person secondarily seek medical treatment for his/her injury? By facility type^a^	All % (n/139)	Minor severity % (n/62)	Major severity % (n/77)
Primary any	53.2 (74)	64.5 (40)	44.2 (34)
Secondary any	45.3 (63)	33.9 (21)	54.5 (42)
Tertiary	0.7 (1)	1.6 (1)	0 (0)
Other	0.7 (1)	0 (0)	1.3 (1)
For those seeking care, how satisfied were they with the care quality?	All % (n/465)	Minor severity % (n/312)	Major severity % (n/153)
Very satisfied	47.3 (221)	51.6 (161)	38.6 (59)
Satisfied	30.0 (140)	27.9 (87)	34.6 (53)
Neither satisfied nor dissatisfied	9.2 (43)	8 (25)	11.8 (18)
Dissatisfied	10.5 (49)	9.6 (30)	12.4 (19)
Very dissatisfied	1.7 (8)	1.9 (6)	0.7 (1)
Don’t know / can’t remember	1.3 (6)	1 (3)	2.0 (3)

^a^Further facility type breakdown available in Additional file 1: Table 3

**Fig. 4:**
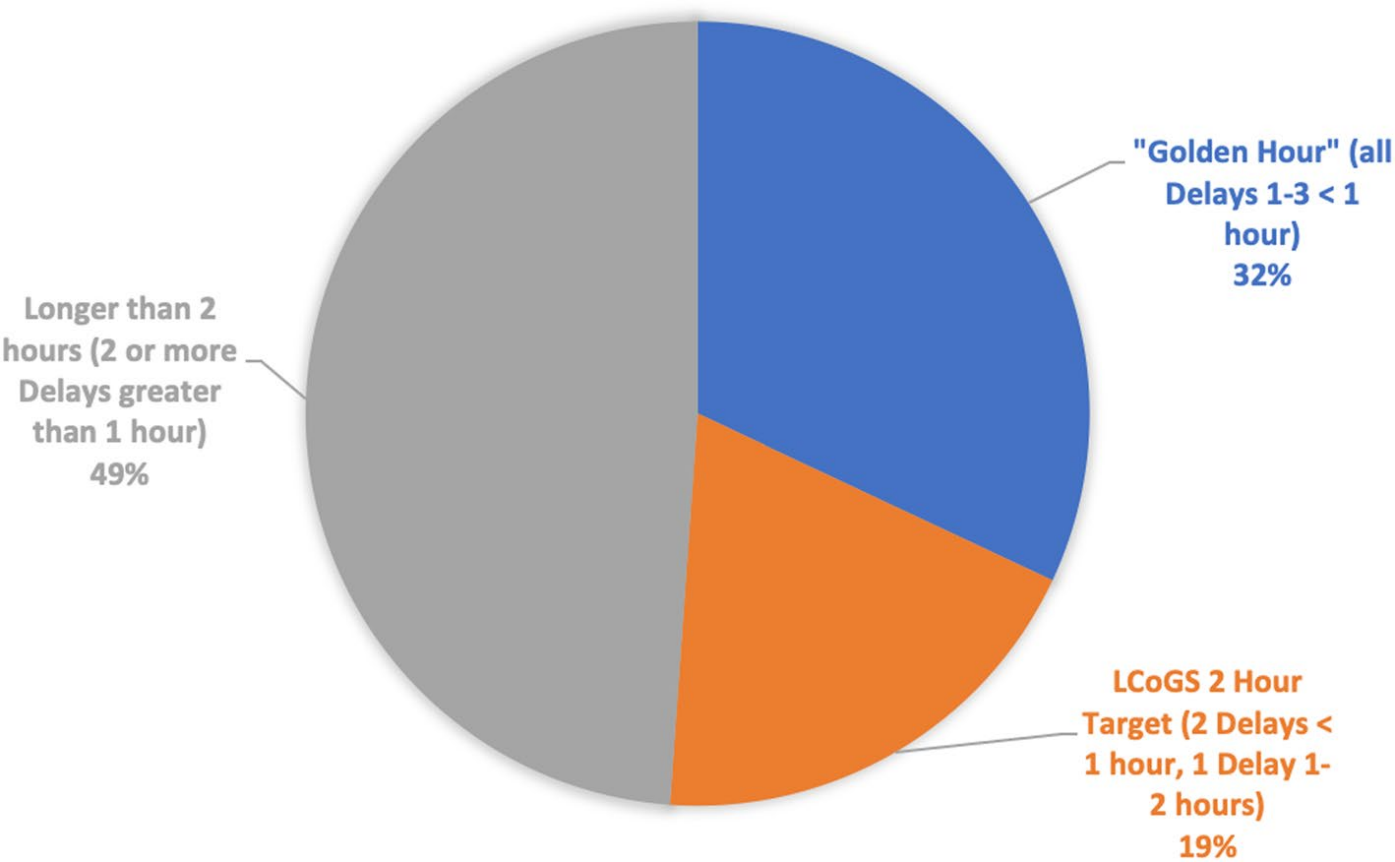
Proportion of patients receiving care within 1 and 2 hours from injury. Those receiving care from time of injury within 1 hour are shown in blue, between 1–2 hours in orange and longer than 2 hour in grey

Almost a third, 32.0% (142/444), of injured patients who sought care, reported less than 1-h delay for each of seeking, reaching, and receiving care (Fig. 4) and therefore received care within the “Golden Hour”. A further 19.1% (85/444) likely received care within the LCoGS 2 h metric. For 48.9% (217/444), care took longer than 2 h from point of injury. Whilst more minor injuries received care within the “Golden Hour” than major injuries, this was not significant (33.2% vs 29.5%, *p* = 0.577) (Additional file 1: Table 1).

For those who sought care, over three quarters, 77.3% (361/467) were either satisfied or very satisfied with the quality of care received and 18.0% (84/467) stayed overnight in a health facility. The median length of overnight stay was two days (IQR 1–3, maximum 33).

On univariable analysis, major injury was associated with being more likely to have; sought care than not (94.4% vs 69.8% *p* < 0.001), attended a second place of care than not (50.3% vs 19.9%, *P* < 0.001), stayed overnight at a facility than not (22.9% vs 15.4% *P* = 0.047). No such difference was seen for which facility type (primary or referral) was attended first (*p* = 0.156) although few people had visited a referral facility first (Additional file 1: Table 2).

The majority, 93.7% (133/142), of those who did not seek care had minor injuries. Reasons given included believing the injury was not serious enough, 60.6% (86/142), having other priorities or responsibilities, 21.8% (31/142), being too difficult to get transport to a health facility, 19% (27/142) and the family member responsible for decisions about seeking care not wanting the injured person to seek care 15.5% (22/142) (Table 2). The stated most important reason was most commonly that the injury was not serious enough 52.1% (74/142), followed by it being too difficult to get transport to a health facility 13.4% (19/142), the financial cost of seeking care being too much 5.6% (8/142) and a preference for traditional healers 4.9% (7/142).

**Table 2:** Reasons given for injured persons not seeking care

	**All % (n/142)**	**Minor % (n/133)**	**Major % (n/9)**
**What were the reasons given for not seeking care (all that apply)?**			
Injury was not serious enough (refused *n* = 1)	60.6 (86)	62.4 (83)	22.2 (2)
Other priorities or responsibilities (refused *n* = 1)	21.8 (31)	22.6 (30)	0 (0)
Too difficult to get transport	19 (27)	18.8 (25)	22.2 (2)
The family member responsible for decisions about seeking care did not want the injured person to seek care (refused *n* = 1)	15.5 (22)	15 (20)	11.1 (1)
The financial cost was too much	11.3 (16)	9.8 (13)	22.2 (2)
The health facility was too far away	11.3 (16)	11.3 (15)	11.1 (1)
Preference for traditional healers	10.6 (15)	11.3 (15)	0 (0)
The health facility would not provide effective treatment	4.9 (7)	4.5 (6)	11.1 (1)
Belief it is not right to seek care following injury (refused *n* = 2)	3.5 (5)	3.8 (5)	0 (0)
The health facility would not treat the injured person with respect	3.5 (5)	3 (4)	11.1 (1)
Other—Self Care—first aid, medication	12.7 (18)	12 (16)	22.2 (2)
**What is the most important reason why the injured person did not seek medical care? (refused = 1)**			
Injury was not serious enough	52.1 (74)	53.4 (71)	22.2 (2)
Too difficult to get transport	13.4(19)	12.8 (17)	22.2 (2)
The financial cost was too much	5.6(8)	4.5 (6)	11.1 (1)
Preference for traditional healers	4.9(7)	5.3 (7)	0 (0)
The health facility would not provide effective treatment	4.2(6)	3.8 (5)	11.1 (1)
Other—Self Care—first aid, medication, unclear	5.6(8)	4.5 (6)	22.2 (2)

Presented here are those reasons with *n* > 5. For the full list see Additional file 1: Table 4

## Discussion

Our household survey confirmed a substantial burden of injuries within our population of study comparable to other rural low-income country settings. We found a community preference to attend primary facilities directly following injury, almost half of whom did not receive care within 2 h of injury. Almost a quarter of those injured in this population did not seek care at all. Although most of these injuries were minor, we identified important health system barriers inhibiting timely access to care.

Our reported injury incidence of 6900 per 100,000 is comparable with that observed in other sub-Saharan African settings ranging from 4300 – 15,200 per 100,000 [22, 38–43]. Injuries were more common in younger [41, 42] or male [38–40, 42] community members. The younger and male sex predominance is a well described global phenomenon possibly driven by alcohol use, dangerous occupations, or risk-taking behaviour [44].

The initial preference for seeking care in primary facilities, even for people with major injuries, confirmed a lack of bypassing to referral facilities. This is consistent with the experience of the injured in Malawi and other LMICs, with the injured often going directly to the nearest healthcare facility, which may not have the capacity to manage trauma patients [45]. For instance, in Tanzania, only 6% of trauma patients had travelled direct to a tertiary facility for their care [46]. Seeking care in primary facilities in our study, as opposed to bypassing to secondary and tertiary care, may simply reflect on the rural context and access to the facility which is closest; as our previous study showed that 94% of this population in Karonga could theoretically reach a primary facility within 1 h and the remaining 6% could access within 2 h [47]. The preference for seeking care in primary facilities in our rurally located study contrasts with what was found in a study from an urban setting in Lilongwe which reported that most patients came directly to the tertiary facility with only 13.1% of trauma cases being referred from other facilities [45]. This care seeking pattern observed in our rural population subsequently necessitates interfacility transfer for those with serious injuries to referral facilities. The lack of rural bypassing to secondary and tertiary care is problematic as it can lead to potentially avoidable delays in injured patients receiving specialist care or intervention not available in primary facilities. Unfortunately, long delays can be common for injured patients requiring transfer in similar contexts. In Tanzania, 85% of traumatic brain injured patients requiring interfacility transfer, were delayed by over 4 h [48]. Whilst in Rwanda, half of referred trauma patients took over 2 days to be transferred [6].

Severe injuries can require life or limb saving time-critical intervention. Long delays before receiving quality facility care can increase the risk of dying following severe injury [49–55]. We found that only one third and one half received care within the golden hour and LCoGS 2 h targets respectively. This estimate is compounded by initial treatment taking place in primary facilities, without capability to undertake the time critical procedures required for more severely injured patients. Although the concept of the golden hour and LCoGS 2 h timeframe may not be relevant for all injuries, they remain a benchmark for guiding trauma system strengthening [36].

Our survey found almost a quarter of those sustaining an injury did not seek care, with lack of care seeking still reasonably high in those with major injuries (at around 6%). Whilst the first delay is under researched in trauma care literature [56] evidence for a real and important phase one delay following trauma in LMICs is growing, with multiple studies providing quantitative or qualitative data. First delay barriers represented two fifths of reasons given for delay following fractures treated at facilities across 18 different LMICs, causing a median 6 day delay which was longer than that attributed to reaching or receiving care [57]. Community based household survey data of extremity injuries in Rwanda found over half did not seek formal medical care [22]. and similar low levels of care seeking following injury have been described in Cameroon [23], China [24], and rural Kenya [25]. In Ghana, not seeking injury care was more pronounced for rural populations than urban [26].

We found that other priorities and responsibilities was a commonly cited reason. This phenomenon likely relates to the health literacy of those community members involved who may underestimate injury severity, whilst also relating to financial concerns such as not wishing to incur financial loss, either through providing transport, losing income or being held liable. Whilst not as prominent in the injury care literature as other barriers, reliance on community members to support care seeking decisions, was shown to act as a barrier to injury care for head injuries in Uganda [58]. Such community decision making was also considered a high priority Delay 1 barrier in a stakeholder mapping workshop in Rwanda [59] and the advice of others played a role in seeking eye injury care in Tanzania [60]. Community empowerment and first aid training has been proposed as a means to support a better bystander prehospital response following injuries in Malawi and similar LMIC settings [46, 61, 62].

In Malawi, direct payment for care is required at private or faith-based facilities [63, 64]. We found, as have others, that these facility types play a lesser role compared to government facilities in the immediate management of injured patients [30, 65]. Most injured patients sought care at government funded facilities which should be free at point of access for Malawian nationals. Nevertheless, cost was found a common and important reason for not seeking care. The role of financial issues in delaying or preventing care access has been found in other sub-Saharan Africa and LMIC settings. Community based surveys from Ghana, Cameroon, Sudan, Rwanda and China found financial reasons for not seeking care were present in between one tenth and a half of cases [22–24, 28, 30]. Other studies have evidenced this effect too. In South Africa, analysis of verbal autopsy data found that costs prohibiting care seeking were associated with one fifth of avoidable trauma deaths [29]. A stakeholder mapping workshop in Rwanda identified a number of financial factors including cost of transport, fear of loss of earnings, fear of impoverishment and cost perceptions, were barriers to seeking injury care [59].

A preference for traditional healers was also an important reason for some not seeking care. This phenomenon is similarly observed elsewhere in Malawi. A tenth of children receiving tertiary burn care in Lilongwe had first tried traditional medicine, which was associated with delay in presentation and increased odds of mortality [66]. The role of traditional healers in providing injury care in some sub-Saharan African contexts can be substantial. In surveys in Ghana and Sudan around a tenth of injured person respondents sought traditional healer care, often as the only provider [28, 30]. Other studies in Ghana and Kenya have found them to be the most common provider choice [25, 67] Traditional healer preference can cause longer delays to seeking formal care than other potential barriers, with an average of eight days in one study [57] However this preference should not be seen in isolation as it has strong mechanistic links with other barriers to seeking care. Decisions for treatment by traditional healers may be taken by someone other than the patient, such as senior family members, as a cultural normative behaviour [67] Some injured people believe traditional medicine to be of better quality or more effective [67], less costly [23, 67] and less fear inducing than attending hospitals [67].

Our study has some limitations. It was limited to a rural population. Urban and rural populations in sub-Saharan Africa experience differing burdens of injury [38, 39]. Future urban comparisons could add perspective to inform more generalisable future preventative and research strategies. Injuries were self-reported and, as typically the case with such an approach [21], it was not possible to independently verify the reported injuries. Self-reporting can also underestimate certain injury types, such as domestic violence [21]. Similarly, the assessment of injury severity was based on self-reported physical disability rather than anatomic or physiological scoring methods [68–72]. This is commonly the case for household surveys since other severity assessment methods require a clinical assessment at the time of injury [21].

Similarly, such surveys are subject to recall bias. Recall loss can occur in community injury household surveys. This can be more pronounced in minor injuries [39, 73, 74]. To compensate some researchers use shorter recall reference frames [40], or extrapolate minor injuries from the most recent recall periods to calculate annual incidence [33]. We used a 12 month recall period to capture more injured patients in the survey and so better understand the health system challenges with seeking, reaching and receiving care. However, our findings likely underestimate the true incidence, particularly of minor injuries, within the population, as recall is likely to be better for major injuries. Using uninjured proxies may exacerbate recall inaccuracy, although this is commonly practised and pragmatic for such surveys [21]. As some injuries are known to be seasonal in comparable contexts, such injuries could have been misrepresented [75].

The survey was limited in its assessment of the barriers to care. Existing barriers were only asked of those who did not seek care. Those who accessed care may still have delayed seeking, the reason for which we would not have captured. However, since our approach focussed on barriers preventing care access, we would likely identify the most potent barriers within the health system. The survey used injury types and mechanisms according to the WHO community survey tool [21].

The household survey could not objectively assess whether the care received was appropriate and effective for the specific injury. Although, most injured patients reported being satisfied or very satisfied with the quality of care received. Patient satisfaction can be influenced by several factors including gratitude, financial cost, ability to access care, short term outcomes and patent expectations [76]. However, patient-reported satisfaction in LMICs does not always correlate with other dimensions of care processes or outcomes and associations with poor technical quality care have even been reported [76]. We did not collect data on occupation, education and socio-economic status, which may have had associations with injury incidence. However, exploring these associations with injury incidence was not an objective of this study where priority was given to questions related to health care seeking and access.

## Conclusion

Injuries in Northern Malawi are substantial and community derived data, such as from our study, is necessary to fully understand the burden of injuries. A substantial proportion of those injured in this population did not seek care. Whilst most injuries were minor, we identified important health system access barriers to address such as competing priorities, access to transport and financial burden. In this context community preference is to attend primary facilities directly following injury, with almost half still not receiving care within 2 h of injury.

### Supplementary Information

**Additional file 1: Table 1.** Patients receiving care within 1 and 2 hours from injury according to injury severity. **Table 2.** Univariable analysis of factors associated with major rather than minor injury severity. **Table 3.** Household survey health facility care seeking by facility type breakdown. **Table 4.** Reasons given for injured persons not seeking care (all responses).

## Data Availability

The datasets used during the current study are available from the corresponding author on reasonable request.

## Abbreviations

LMIC: Low and Middle Income Country
LCoGS: Lancet Commission on Global Surgery
WHO: World Health Organisation
MEIRU: Malawi Epidemiology and Intervention Research Unit
HDSS: Health and Demographic Surveillance System
DALY: Disability Adjusted Life Years
CHAM: Christian Health Association of Malawi
