# Supplementary material for: Access to quality care after injury in Northern Malawi: results of a household survey
Source: BMC Health Serv Res. 2024 Jan 24;24:131. doi: 10.1186/s12913-023-10521-8 (PMC10809521; doi:10.1186/s12913-023-10521-8)
Supplement: Supplementary file 1 — Additional file 1: Table 1. Patients receiving care within 1 and 2 hours from injury according to injury severity. Table 2. Univariable analysis of factors associated with major rather than minor injury severity. Table 3. Household survey health facility care seeking by facility type breakdown. Table 4. Reasons given for injured persons not seeking care (all responses). [file 12913_2023_10521_MOESM1_ESM.docx]

Additional file 1: Table 1 Patients receiving care within 1 and 2 hours from injury according to injury severity.

|  | All % (n/444) | Minor % (n/295) | Major % (n/149) | P Value |
| --- | --- | --- | --- | --- |
| **"Golden Hour"** (all Delays 1-3 < 1 hour) | 32.0 (142) | 33.2 (98) | 29.5 (44) | 0.577 |
| **LCoGS 2 Hour Target but not “Golden Hour”** *(2 Delays < 1 hour, 1 Delay 1-2 hours)* | 59.0 (85) | 19.7 (58) | 18.1 (27) |  |
| **Longer than 2 hours** (2 or more Delays greater than 1 hour) | 48.9 (217) | 47.1 (139) | 52.3 (78) |  |
| “Don’t know” for any answer counted as missing. | | | | |

Additional file 1: Table 2 Univariable analysis of factors associated with major rather than minor injury severity.

|  | All  % (n/611) | Minor  % (n/447) | Major  % (n/162) | P value |
| --- | --- | --- | --- | --- |
|  |  |  |  |  |
| Formal care-seeking |  |  |  |  |
| Yes | 76.4 (465) | 69.8 (312) | 94.4 (153) | <0.001 |
| No | 23.2 (142) | 29.8 (133) | 5.6 (9) |  |
| Don’t know | 0.2 (1) | 0.2 (1) | 0 (0) |  |
| Missing | 0.2 (1) |  |  |  |
|  |  |  |  |  |
| For those seeking care, did they stay in a facility overnight? | (n/465) | (n/312) | (n/153) |  |
| Yes | 17.8 (83) | 15.4 (48) | 22.9 (35) | 0.047 |
| No | 82.2 (382) | 84.6 (264) | 77.1 (118) |  |
|  |  |  |  |  |
| Did the injured person attend a second place of care following the injury? |  |  |  |  |
| No | 70.1 (326) | 80.4 (250) | 49.7 (76) | <0.001 |
| Yes | 29.9 (139) | 19.9 (62) | 50.3 (77) |  |
|  |  |  |  |  |
| First Facility Type | (n/464) | (n/312) | (n/153) |  |
| Primary | 96.6 (448) | 95.6 (297) | 98.7 (151) | 0.156 |
| Referral | 2.2 (10) | 2.6 (8) | 1.3 (2) |  |
| Traditional Healer | 1.3 (6) | 1.9 (6) | 0 |  |

Additional file 1: Table 4 Reasons given for injured persons not seeking care (all responses).

| **What were the reasons given for not seeking care (all that apply)?** | **All % (n/142)** | **Minor % (n/133)** | **Major % (n/9)** |
| --- | --- | --- | --- |
| Injury was not serious enough (refused n = 1) | 60.6 (86) | 62.4 (83) | 22.2 (2) |
| Other priorities or responsibilities (refused n = 1) | 21.8 (31) | 22.6 (30) | 0 (0) |
| Too difficult to get transport | 19 (27) | 18.8 (25) | 22.2 (2) |
| The family member responsible for decisions about seeking care did not want the injured person to seek care (refused n = 1) | 15.5 (22) | 15 (20) | 11.1 (1) |
| The financial cost was too much | 11.3 (16) | 9.8 (13) | 22.2 (2) |
| The health facility was too far away | 11.3 (16) | 11.3 (15) | 11.1 (1) |
| Preference for traditional healers | 10.6 (15) | 11.3 (15) | 0 (0) |
| The health facility would not provide effective treatment | 4.9 (7) | 4.5 (6) | 11.1 (1) |
| Belief it is not right to seek care following injury (refused n = 2) | 3.5 (5) | 3.8 (5) | 0 (0) |
| The health facility would not treat the injured person with respect | 3.5 (5) | 3 (4) | 11.1 (1) |
| Did not know healthcare was available | 2.1 (3) | 2.3 (3) | 0 (0) |
| People fear the consequences of helping an injured person e.g. being accused of causing the injury | 1.4 (2) | 0.8 (1) | 11.1 (1) |
| The health facility would not communicate effectively (don’t know n = 1) | 0 | 0 (0) | 0 (0) |
| Other - Self Care - first aid, medication | 12.7 (18) | 12 (16) | 22.2 (2) |
| Other - Heard no clinician available | 2.1 (3) | 2.3 (3) | 0 (0) |
| Other - Alone, no-one to assist | 1.4 (2) | 1.5 (2) | 0 (0) |
| Other - Out of Hours | 1.4 (2) | 1.5 (2) | 0 (0) |
| Other - Afraid of treatments | 0.7 (1) | 0.8 (1) | 0 (0) |
| Other - Dislike for seeking care | 0.7 (1) | 0.8 (1) | 0 (0) |
| Other - Health workers don’t respond in right manner | 0.7 (1) | 0 (0) | 11.1 (1) |
| Other - Negative previous facility experience | 0.7 (1) | 0.8 (1) | 0 (0) |
| Other - Mental health problem | 0.7 (1) | 0 (0) | 11.1 (1) |
| **What is the most important reason why the injured person did not seek medical care? (refused = 1)** |  |  |  |
| Injury was not serious enough | 52.1 (74) | 53.4 (71) | 22.2 (2) |
| Too difficult to get transport | 13.4(19) | 12.8 (17) | 22.2 (2) |
| The financial cost was too much | 5.6(8) | 4.5 (6) | 11.1 (1) |
| Preference for traditional healers | 4.9(7) | 5.3 (7) | 0 (0) |
| The health facility would not provide effective treatment | 4.2(6) | 3.8 (5) | 11.1 (1) |
| Belief it is not right to seek care following injury | 2.1(3) | 2.3 (3) | 0 (0) |
| The family member responsible for decisions about seeking care did not want the injured person to seek care | 2.1(3) | 2.3 (3) | 0 (0) |
| Other priorities or responsibilities | 1.4(2) | 1.5 (2) | 0 (0) |
| Did not know healthcare was available | 1.4(2) | 1.5 (2) | 0 (0) |
| The health facility was too far away | 1.4(2) | 1.5 (2) | 0 (0) |
| The health facility would not treat the injured person with respect | 1.4(2) | 1.5 (2) | 0 (0) |
| People fear the consequences of helping an injured person, e.g. being accused of causing the injury | 0.7(1) | 0 () | 0 () |
| Other - Self Care - first aid, medication, unclear | 5.6(8) | 4.5 (6) | 22.2 (2) |
| Other - Afraid of treatments | 0.7(1) | 0.8 (1) | 0 (0) |
| Other - Alone, no-one to assist | 0.7(1) | 0.8 (1) | 0 (0) |
| Other - Heard no clinician available | 0.7(1) | 0.8 (1) | 0 (0) |
| Other – Mental Health Problem | 0.7(1) | 0 (0) | 11.1 (1) |

**Additional file 1: Table 3 Household survey health facility care seeking by facility type breakdown.**

| Where did the injured person first seek medical treatment for his/her injury? By facility type | All % (n/465) | Minor severity %(n/311) | Major severity %(n/153) |
| --- | --- | --- | --- |
| Primary any | 96.3 (448) | 95.2 (296) | 98.7 (151) |
| Government Primary | 67.5 (314) | 65.9 (205) | 71.2 (109) |
| Private Primary | 13.1 (61) | 13.5 (42) | 12.4 (19) |
| Faith-Based Primary | 11.6 (54) | 12.2 (38) | 9.8 (15) |
| Military Primary | 4.1 (19) | 3.5 (11) | 5.2 (8) |
| Secondary any | 1.9 (9) | 2.3 (7) | 1.3 (2) |
| Government Secondary | 1.9 (9) | 2.3 (7) | 1.3 (2) |
| Tertiary | 0.2 (1) | 0.3 (1) | 0 (0) |
| Other | 1.5 (7) | 2.3 (7) | 0 (0) |
| Traditional Healer | 1.3 (6) | 1.9 (6) | 0 (0) |
| Vet | 0.2 (1) | 0.3 (1) | 0 (0) |
|  |  |  |  |
| Where did the injured person secondarily seek medical treatment for his/her injury? By facility type | All % (n/139) | Minor severity % (n/62) | Major severity % (n/77) |
| Primary any | 53.2 (74) | 64.5 (40) | 44.2 (34) |
| Government Primary | 22.3 (31) | 30.6 (19) | 15.6 (12) |
| Private Primary | 20.9 (29) | 24.2 (15) | 18.2 (14) |
| Faith-Based Primary | 7.9 (11) | 9.7 (6) | 6.5 (5) |
| Military Primary | 2.2 (3) | 0 (0) | 3.9 (3) |
| Secondary any | 45.3 (63) | 33.9 (21) | 54.5 (42) |
| Government Secondary | 44.6  (62) | 33.9 (21) | 53.2 (41) |
| FB Secondary | 0.7 (1) | 0 (0) | 1.3 (1) |
| Tertiary | 0.7 (1) | 1.6 (1) | 0 (0) |
| Other | 0.7 (1) | 0 (0) | 1.3 (1) |
| Traditional Healer | 0.7 (1) | 0 (0) | 1.3 (1) |
|  |  |  |  |
